# Editor’s Book Table

**Published:** 1871-05

**Authors:** 


### Editor's Book Table.

[NOTE.—All works reviewed in the columns of the CHICAGO MEDICAL JOURNAL may be found in the extensive stock of W. B. KEEN & COOKE, whose catalogue of Medical Books will be sent to any address upon request.]

BOOKS RECEIVED.

*Chemistry: General, Medical and Pharmaceutical, including the Chemistry of the U. S. Pharmacopœia. A Manual on the General Principles of the Science and their Applications to Medicine and Pharmacy.* By JOHN ATTFIELD, Ph. D., F. C. S., Professor of Practical Chemistry to the Pharmaceutical Society of Great Britain, etc., etc. From the second and enlarged English edition. Revised by the Author. Philadelphia: Henry C. Lea. 1871. Pp. 552.

A very convenient manual for students of medicine or those engaged in practical pharmacy. A comprehensive index, containing 5,000 references, gives every facility for speedy consultation and investigation of any desired topic.

*Minnesota as a Home for Invalids.* By BREWER MATTOCKS, M.D., President of the Board of Health, St. Paul; Physician to St. Joseph's Hospital. Philadelphia: J. B. Lippincott & Co.; St. Paul, Minn., D. D. Merrill, Randall & Co. 1871. Pp. 200. From Cobb Brothers, Lake street.

The author aims to present to the public, so far as able, the views of the medical profession of the State of Minnesota as to its advantages as a climate for invalids. The meteorological observations are those reported for the Smithsonian Institute. So much has been said about Minnesota as a place of exile for a certain class of invalids, that this little book is both seasonable and valuable, and should be in the hands of both physicians and patients interested in the subject.

*Insanity and its Treatment. Lectures on the Treatment, Medical and Legal, of Insane Patients.* By G. Fielding Blandford, M. D., Oxon., etc., etc. With a Summary of the Laws in force in the United States on the Confinement of the Insane. By Isaac Ray, M.D. Philadelphia: Henry C. Lea. 1871. Pp. 471.

We can recommend this work in warm terms as containing in compact form and concise terms a large amount of valuable information. The Appendix by Dr. Ray is practically useful. It is some consolation to find that Illinois is not alone in the possession of atrocious usages necessarily sequent to atrocious laws regarding the commitment of the insane. When, O when! will the people cease to send their supremest donkeys to be their legislators?

*U. S. Sanitary Commission Memoirs.* Surgical. Second Vol. Edited by Prof. Frank Hastings Hamilton. New York: Published for the United States Sanitary Commission, by Hurd & Houghton. Cambridge: Riverside Press. 1871. Pp. 580, and chromo-lithographs.

The first surgical volume of this series was noticed last year, (Journal, 1870, p. 436.) The present volume contains:

I. Analysis of 439 Recorded Amputations in the Contiguity of the Lower Extremity. By Stephen Smith, M.D.

II. Investigations upon the Nature, Causes and Treatment of Hospital Gangrene, as it prevailed in the Confederate Armies, 1861–5. By Joseph Jones, M.D., Professor of Chemistry in the Medical Department of the University of Louisiana, New Orleans. Formerly Surgeon in the Provisional Army of the Confederate States.

### PAMPHLETS.

*Proceedings of the American Pharmaceutical Association, at the Eighteenth Annual Meeting held in Baltimore, Md., September, 1870. Also the Constitution and Roll of Members.* Philadelphia: Sherman & Co., printers. 1870. Pp. 352.

Copies of this quite interesting and valuable volume can be procured of Mr. Louis Strehl, 62 State street, Chicago, who is the State agent for the association.

*Report of the Special Committee of the Medical Society of the District of Columbia, upon the Claims of Homœopaths and other Irregular Practitioners for Professional Recognition in the Medical Service of the U. S. Government, and the Charges brought by the Homœopaths against the U. S. Commission of Army and Navy Pensions.* Published by Resolution of the Society.

*Twenty-Second Annual Announcement of the Woman's Medical College of Pennsylvania,* 1871–2.

*Tenth Annual Report of the Board of Managers of the Woman's Hospital of Philadelphia, Jan.* 1871.

*The Study of Dermatology.* By Louis A. Duhring, M.D., Physician to the Dispensary for Skin Diseases. Philadelphia. Pp. 12.

*Atlanta Medical and Surgical Journal.* Edited by W. F. Westmoreland, M.D., Professor of the Principles and Practice of Surgery in the Atlanta Medical College, and J. C. Westmoreland, M.D., Professor of Materia Medica and Therapeutics in the Atlanta Medical College. April, 1871.

This is numbered as the first of vol. nine, it being, if we mistake not, the third resuscitation of this Journal. We wish better luck for it hereafter. Published monthly, at $3 a year.

*The Virginia Clinical Record.* A monthly Journal of Med. Surg. and Collateral Sciences. M. W. Hazlewood, publisher, Richmond, Va. $1 per annum.

*American Journal of Microscopy.* Devoted to the Elucidation of Scientific and Popular Microscopy. E. M. Hale, M.D., editor; G. Mead & Co., publishers and proprietors, 182 Clark street, Chicago. $2 per annum; single copies, 25 cents.

Several months since, a periodical under this title was started in small quarto form, rather cheaply gotten up. Its success, however, was such as to encourage the publishers to put it in better shape. The result, is the neat monthly now before us, which we take pleasure in recommending to the patronage and support of all interested in the subject.

*M. or N. Similia Similibus Curantur.* By J. G. WHYTTE MELVILLE, author of Kate Coventry, etc., etc. New York: Leypoldt, Holt & Williams, 25 Bond st. 1871. Pp. 159.

This is not a medical work as its motto would suggest, but an effort to show that a wound by one of Dan Cupid's arrows can be cured by another. The illustration in its way is successful. The old love is cured by a new one, as an old suit of clothes is well replaced by a later fashion and make; or as the loss of an old five-dollar greenback is materially alleviated by finding a spick and span new one of the same or a higher denomination.

*Fertility of the Native Americans of the Present Generation.* By H. O. HITCHCOCK, M.D., of Kalamazoo, Mich. Read before the Kalamazoo Med. Association, March 14, 1871.

This is one of the best arguments we have yet seen attempting to prove that the Native American (not Aboriginal) stock is wanting, and constantly diminishing in fertility. Dr. H. is not the first who has lugubriously exclaimed, *O Tempora! O Mores!* Not the first who has mourned (and proved) the degeneracy ot these latter days.

[Privately—in spite of our Lowell namesake's essays, and even this now before us, we don't believe a word in the doctrine—Hush—h!]

